# The End Justifies the Means: Chagas Disease from a Perspective of the Host–*Trypanosoma cruzi* Interaction

**DOI:** 10.3390/life14040488

**Published:** 2024-04-09

**Authors:** Izadora Volpato Rossi, Denise Andréa Silva de Souza, Marcel Ivan Ramirez

**Affiliations:** 1 Graduate Program in Microbiology, Parasitology and Pathology, Federal University of Paraná, Curitiba 81531-980, PR, Brazil; irossi@aluno.fiocruz.br; 2 Laboratory of Cell Biology, Carlos Chagas Institute/Oswaldo Cruz Foundation (FIOCRUZ-PR), Curitiba 81310-020, PR, Brazil; desouza@aluno.fiocruz.br

**Keywords:** *Trypanosoma cruzi*, Chagas disease, pathogenesis, host–pathogen interaction, tropism

## Abstract

The neglected Chagas disease (CD) is caused by the protozoan parasite *Trypanosoma cruzi*. Despite CD dispersion throughout the world, it prevails in tropical areas affecting mainly poor communities, causing devastating health, social and economic consequences. Clinically, CD is marked by a mildly symptomatic acute phase, and a chronic phase characterized by cardiac and/or digestive complications. Current treatment for CD relies on medications with strong side effects and reduced effectiveness. The complex interaction between the parasite and the host outlines the etiology and progression of CD. The unique characteristics and high adaptability of *T. cruzi*, its mechanisms of persistence, and evasion of the immune system seem to influence the course of the disease. Despite the efforts to uncover the pathology of CD, there are many gaps in understanding how it is established and reaches chronicity. Also, the lack of effective treatments and protective vaccines constitute challenges for public health. Here, we explain the background in which CD is established, from the peculiarities of *T. cruzi* molecular biology to the development of the host’s immune response leading to the pathophysiology of CD. We also discuss the state of the art of treatments for CD and current challenges in basic and applied science.

## 1. Introduction

Chagas disease (CD), or American trypanosomiasis, is a zoonotic disease caused by the protozoan parasite *Trypanosoma cruzi*. The classic transmission of *T. cruzi* is maintained by insect vectors (triatomines of the genera *Triatoma*, *Panstrongylus*, *Rhodnius*) and domestic and wild mammals that serve as reservoirs. The vectors become infected by feeding on the blood of an infected host (including humans and other mammals). In the gastrointestinal tract of insects, these forms evolve into epimastigotes and then into metacyclic trypomastigotes. When the vector takes a blood meal, it releases these trypomastigotes in the excreta and the parasites penetrate the wound into intact mucous membranes, such as the ocular conjunctiva. Once inside the host, trypomastigotes invade the cells and maintain the infection as amastigotes [1,2]. In addition to vector transmission, in recent decades outbreaks of oral transmission of CD have been frequently described and can reach up to 70% of cases in some regions [2]. These outbreaks were associated with the consumption of contaminated food/drinks such as bushmeat, vegetables, sugar cane extract, and açaí pulp, among others [3]. The mortality rate in patients infected orally is reported to be higher (8–35%) when compared to classical vector transmission through vector excreta (<5–10%) [4]. Other routes of transmission include blood transfusion or organ transplantation from infected donors, and mother-to-child (congenital) transmission [5].

According to the World Health Organization [6], CD is among the 20 neglected tropical diseases (NTD), which are diseases that prevail in tropical areas, mainly affecting poor communities and causing devastating health, social and economic consequences for many individuals. Chagas disease is endemic in 21 countries in Latin America and has traditionally been confined to poor rural areas in Central and South America, but has spread to other regions of the world due to migratory flow [1].

In 1909, Carlos Ribeiro Justiniano das Chagas, a Brazilian medical researcher, observed in a two-year old girl called Berenice symptoms of an unknown disease. In a brilliant effort for understanding the disease, he identified the etiological agent of CD, the protozoan *T. cruzi*, its hosts, both the triatomine vectors (mainly *Triatoma infestans*, *T. dimidiate*, and *Rhodnius prolixus*) and the mammalian reservoirs, the different stages of development of the parasite, as well as the clinical aspects of CD [7]. However, even after more than one century since his discovery, the treatment of CD relies only on two nitroheterocyclic drugs developed more than 50 years ago: nifurtimox (NFX) and benznidazole (BZN). These drugs have serious disadvantages including long treatment periods, toxic side effects and reduced efficacy in the chronic phase [8].

The clinical course of CD generally comprises an acute and a chronic phase. The acute phase is generally mildly symptomatic (with common clinical signs such as fever, headache, and diarrhea) and fewer patients presenting lymphadenopathy, hepatosplenomegaly, myocarditis, pericardial effusion and heart failure or meningoencephalitis. Parasitemia is evident in the beginning of the infection and lasts up to three months, when infected individuals progress to a chronic phase [1]. While many patients remain in an undetermined phase for years or decades, i.e., without clinical symptoms but positive serology, approximately 30% of infected individuals progress to clinically relevant CD. Chagas disease includes a wide spectrum of manifestations, ranging from myocardial involvement (with left ventricular systolic dysfunction, dilated cardiomyopathy, arrhythmias, thromboembolic events, and terminal heart failure) to gastrointestinal manifestations (such as megaesophagus and megacolon) [9]. In 2020, the WHO introduced a roadmap with the following objectives: verifying the interruption of vector-borne transmission, verifying the interruption of transmission through transfusions and organ transplants, eliminating congenital CD, and expanding the coverage of antiparasitic treatment in the population at risk [6].

Despite being an ancient disease that has seen a decrease in infection due to apparent vector control and the urbanization of society, new migratory flows in North America and Europe, as well as incidences of oral contamination, have revitalized the discussion of policies for the diagnosis and control of the disease [10]. Despite the efforts of the scientific community, understanding CD pathophysiology remains challenging. Many mechanisms appear to shape the etiology of CD, and it is now known that complex interactions between host and parasite outline the course of infection. Furthermore, the wide genetic and phenotypic variability of different *T. cruzi* strains, and its invasion mechanisms and virulence factors, allow its persistence for long years in the host. Here, we discuss the main factors that contribute to the pathogenesis of CD, from the molecular peculiarities of the parasite and its virulence factors to pathogen–host interactions and the immune response.

## 2. *Trypanosoma cruzi* Has a Peculiar Molecular Biology

*Trypanosoma cruzi* presents extraordinary genetic diversity; currently, based on genetic, biochemical and biological markers, the *T. cruzi* population is divided into seven genetic lineages or discrete typing units (DTUs), named TcI to TcVI [11] and TcBat, which is restricted to bats [12]. Another classification has described the intraspecific variation in *T. cruzi* based on mitochondrial sequences, and three clades were defined, presenting some similarities with the DTU classification: clade A corresponds to TcI, clade B corresponds to the TcIII, TcIV, TcV and TcVI strains, and clade C corresponds exclusively to TcII [13].

It is well-known that *T. cruzi* pathogenicity varies greatly between strains, even within DTUs, raising questions about the reliability of the *T. cruzi* classification and if it should be considered a complex of species rather than a unique species [14]. The chromosome length is a good example of the genome complexity reaching the strain level, varying between strains of the same DTU, strains of distinct DTUs, and even clones from the same strain [15].

Genomic plasticity, which can include entire chromosomes or gene deletion/duplication, is an important tool in response to environmental changes like the ones faced by parasites. Aneuploidy, which refers to an unequal number of chromosomal copies, is an ancestral characteristic in trypanosomatids being present in several species of this family, including *Leishmania* spp., *T. cruzi*, and the basal *Paratrypanosoma confusum* [16]. However, aneuploidy later evolved to be almost absent in *T. brucei* and closely related species (*T. congolense* and *T. vivax*) [16]. The peculiarities of *T. brucei* genomic organization may have contributed to this loss, since this parasite presents a larger chromosome size, and hence an increased fitness cost of aneuploidy (i.e., a higher number of genes per chromosome leads to an increased number of genes in an unbalanced proportion). The exceptions are the genes related to the ancestral chromosomal duplication (named Duplicated31) containing housekeeping genes that were also maintained in *T. brucei*. It is interesting to note in this parasite and *T. cruzi*, the Duplicated31 is enriched in genes involved in glycosylation and surface-protein anchoring, i.e., during evolution these parasites transferred to Duplicated31 genes that are crucial for host–pathogen interaction, since both parasites require GPI-anchored proteins for cellular invasion and immune evasion [16].

Within chromosomes, telomeric regions are naturally prone to mutations due their location at the final ends of linear chromosomes, where DNA can be lost during replication. Telomere DNA consists of guanine-rich tandemly repeated double-stranded satellite sequences with a short single-stranded portion at the 3′ end of the chromosome that forms the G-overhang [17]. Several proteins associate to the G-overhang to form the telomere, protecting the chromosome from degradation. In *T. cruzi*, telomeric and subtelomeric regions present a high rate of DNA recombination and a high incidence of multigene families, like trans-sialidases, mucins and MASPs (mucin-associated proteins) [15]. These proteins are crucial for host–parasite interaction, suffering strong evolutionary pressure; therefore, its expansion as a multigene family in regions that are favorable to recombination, i.e., telomeres, is not surprising. Similarly, sub-telomeric regions appear to be involved in the process called VSG (variant surface glycoprotein) switching in *T. brucei* that is the primary immune evasion tool for this parasite [15]. A recent study, analyzing the genome of two *T. cruzi* strains using long-read sequencing technology, shows that the *T. cruzi* genome is organized in a compartmentalized way, being the core compartment characterized by conserved and hypothetical genes in synteny with *Leishmania* spp. and *T. brucei* genomes, and the disruptive compartment comprising rapidly evolved multigene families (like trans-sialidases, MASPs, mucins, among others) [18]. The organization of the core and disruptive regions forms three-dimensional chromatin compartments with different levels of DNA methylation, nucleosome positioning, and chromatin interactions. For this reason, it is proposed that epigenetic mechanisms affect the dynamics of gene expression in *T. cruzi* [19].

Trypanosomatids lack the canonical signals for RNA polymerase II (RNA pol II) promoters; therefore, their transcription is polycistronic. Usually, protein-coding genes have unrelated predicted functions, being separated by short intergenic regions. In the absence of promoters, the transcription by RNA pol II is initiated bi-directionally between two divergent gene clusters and produces a polycistronic pre-mRNA [20]. To produce individual mRNAs, trypanosomes use trans-splicing and polyadenylation simultaneously; i.e., in a polycistronic pre-mRNA, considering two tandem mRNAs, the first mRNA is polyadenylated at its 3′ end right after the second mRNA receives the 5′ cap containing the SL (spliced leader) sequence (trans-splicing) [21]. Interestingly, the SL gene is the only gene transcribed by the RNA pol II that has promoters and terminators, coding for the conserved 39 nucleotides SL cap [20]. However, no sequence for polyadenylation or SL addition has been found in trypanosomatids, with the assumption that polypyrimidine-rich regions within intergenic regions guide SL addition and polyadenylation in these parasites [22].

In the absence of promoters, the regulation of gene expression is mainly at post-transcriptional level, where the 3′ untranslated regions (3′ UTRs) of mRNAs play a crucial role [23]. The continuous supply of transcripts is an important evolutionary gain for trypanosomes, because, despite being energetically consuming, the availability of numerous and different transcripts allows a rapid response to the environmental changes faced by digenetic trypanosomes like *T. cruzi* [21]. The post-transcriptional control of gene expression in trypanosomes is mediated by RNA-binding proteins (RBPs) that control mRNA stability, degradation and the access to the translation machinery [24]. The mRNA pool can also be compartmentalized in P-bodies (processing bodies) or stress granules where transcripts stay stable and ready to be either degraded or translated depending on stress signals [25].

At DNA level, protein abundance is determined by gene duplications. *Trypanosoma cruzi* presents several multigene families, some with hundreds of members, the majority coding for surface proteins that are important for parasite invasion and host immune evasion like the trans-sialidases, MASPS and mucins [15]. Details of the main molecules encoded by multigene families are described in the following topics. 

### 2.1. Trans-Sialidases Family

Trans-sialidase catalyzes the transfer of sialic acid from host cells to mucins present in the membrane of the parasite, protecting them from the host immune system and facilitating cell invasion. Trans-sialidases have a glycosylphosphatidylinositol anchor (GPI-anchor) attaching them to the membrane surface of metacyclic and bloodstream trypomastigotes, and onto the surface of the intracellular amastigotes. Also, TSs can be released to the extracellular milieu via microvesicles, being an important virulence factor [26]. 

The TS family (TS) is the largest multigene family in *T. cruzi*, comprising about 1430 genes [27] that can be divided into eight groups according to the most recent classification based on sequence cluster analysis [28]. The TS groups are defined by specific motifs conferring specialized activities, although not all members of each group present the same functions. For example, some TSs from Group I (like SAPA, TCNA, and TS-epi) have an active trans-sialidase function, whereas others do not present catalytic sites. Some members of Group II (ASP-2, Tc-85, SA85, GP82, and GP90) are involved in host cell attachment and invasion, while others (like FL160) are involved in complement system inhibition [15]. The presence of these eight TS groups in different *T. cruzi* strains was analyzed by Callejas-Hernandéz et al. [29] and the results revealed that different strains have different sets of trans-sialidases that may lead them to be more or less apt to evade the host immune system. In fact, some studies suggest that diverse genetic characteristics of *T. cruzi* may influence the clinical outcome of Chagas disease, like tissue tropism, virulence, drug response, etc. [30]. In this sense, a study using phage display shows that the FLY domain (VTVxNVxLYNR), present in 371 members of the TS family (among them the GP85/TS subfamily expressed in bloodstream trypomastigotes), has different patterns for binding to diverse organs, resembling the tissue tropism found in patients and animal models [31]. Recently, the lysosomal protein LAMP-2 was identified as the host cell receptor for the TS GP82 of *T. cruzi* [32]. Metacyclic parasites adhere to LAMP-2 present at low levels at the host plasma membrane, triggering the lysosome scattering to the cell periphery, thus increasing the availability of LAMP-2 and its binding to the GP82, and therefore promoting the metacyclic internalization in a vacuole formed by the fusion of lysosomes with the plasma membrane [32]. The TS GP82 is also important to *T. cruzi* infection by oral route, since it binds to the gastric mucin [33].

The levels of expression of these genes greatly differ between the TS groups and in the different stages of the parasite’s life cycle. As previously discussed, the involvement of the 3′ UTRs in the control of gene expression is crucial for the rapid response of *T. cruzi* to changes in the environment, like the transition between insect and mammalian hosts. Some correlations were found between the expression profile and characteristics of the 3′ UTRs in genes from the TS family. For example, SAPA and TCNA genes, coding for active trans-sialidases expressed in trypomastigotes, have almost identical 3′ flanking regions [28]. The fine control of the expression of genes related to parasite survival, like the ones involved in host immune evasion and cellular invasion, were also described for other gene families like mucins.

### 2.2. Mucins Family

Mucins are the most abundant glycoproteins present on the *T. cruzi* trypomastigote surface. Mucins are decorated with oligosaccharides O-linked to serine and/or threonine residues, have a GPI-anchor and can be sialylated by active TSs [34]. This is the third largest gene family in *T. cruzi*, comprising 850 genes [27] that can be divided into TcMUC (*T. cruzi* Mucin-like genes) and TcSMUG (*T. cruzi* Small Mucin-like genes). The TcMUC genes are exclusively expressed in the mammalian stages of the parasite, whereas TcSMUG are only expressed in the insect stages. The TcMUCs are crucial for escaping the mammalian immune system, and to promote cell adhesion and invasion [35]. TcMUCs are subdivided into three classes (TcMUC I, II, and III) and contain a signal peptide, a GPI-anchor and a central region that contains a hypervariable section (HV) [15]. TcMUC I are abundant in amastigotes, whereas TcMUC II are predominant in lipid rafts on the membrane of bloodstream trypomastigotes [26]. The GPI-anchor is related to elicit a proinflammatory response by binding to the Toll-like receptor 2 (TLR2) of macrophages [36]. 

TcSMUGs are less diverse than TcMUCs, possibly because they are expressed only in the insect stages of the parasite where they do not suffer the strong evolutive pressure exerted by the mammalian immune system. TcSMUGs can be divided into two groups named L (Large) and S (Small) mucins [37]. The most studied TcSMUG S is the GP35/50 that are expressed in metacyclics and epimastigotes, but they show different functions: in metacyclics, GP35/50 binds to target cells and elicits a Ca^2+^ response that leads to cell invasion; in epimastigotes, GP35/50 is related to the protection from proteases in the insect intestinal tract [38]. The importance of post-transcriptional regulation on the expression of TcSMUG genes was gracefully described by Di Noia and collaborators [37] who found differences in the 3′ UTR of the transcripts between L and S groups, where an AU-rich region in the 3′ UTR is a destabilizing element of the mRNA, downregulating the L group mRNA levels during the transition from epimastigotes to metacyclic trypomastigotes [37]. The different sets of mucin genes expressed during the transition from the insect-dwelling to mammal-dwelling stages shows their importance for parasite differentiation [39] and survival [36], therefore contributing to the pathogenesis of Chagas disease. 

### 2.3. Mucin-Associated Surface Proteins Family (MASPs)

This gene family is named Mucin-Associated Surface Proteins because it encodes proteins that are in close proximity with mucins on *T. cruzi* plasma membrane, and also shares similarities in structure. MASPs contain a signal peptide and a GPI-anchor, and have a variable central region usually with repeated motifs [40]. The MASPs comprise the second largest gene family in the *T. cruzi* genome comprising about 1300 genes [27] with a highly heterogeneous coding region, although MASP mRNAs present a conserved 5′ and 3′ UTRs [40]. 

MASPs are overexpressed in the infective stages of the parasite (metacyclic and bloodstream trypomastigotes), being involved in host cell invasion, as was described by De Pablos and collaborators [41] studying MASP52. Recently, MASP49 was shown to bind to the C-type lectin receptor (mMGL) of murine peritoneal macrophages [42], contributing to cell invasion. Also, MASPs overexpression on the amastigotes membrane before division suggests that these proteins can play a role in the survival and multiplication of the intracellular amastigotes [43]. The differences found in the gene expression between the stages of the *T. cruzi* life cycle reflect the high regulation of this multigene family. 

**Figure 1:**
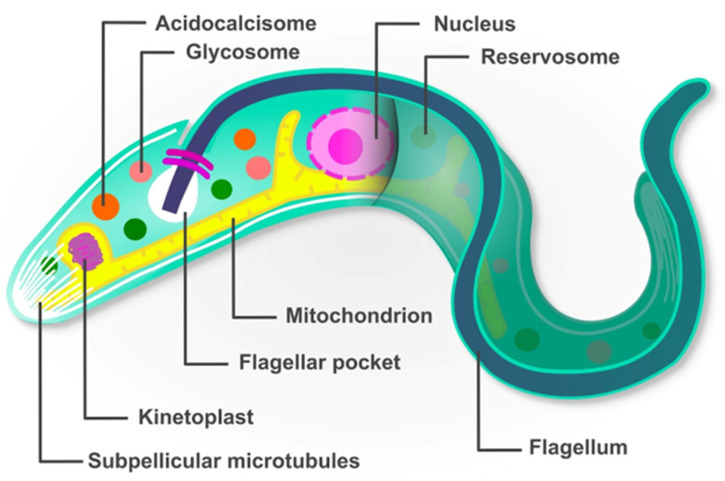
Schematic representation of the unique morphology of *Trypanosoma cruzi*.

The studies performed to date revealed that the plasticity of *T. cruzi*’s genome to generate multiple variants of proteins (like TSs, mucins, and MASPs) through gene duplication, recombination, and mutation, are a source for great antigenic diversity, increasing the parasite fitness and survival by promoting the evasion of the mammalian host immune system. In addition to the complexity given by the repertoire of surface proteins, the parasite’s cellular biology is also unique. *Trypanosoma cruzi* contains organelles shared only between other kinetoplastids. An overview of these features is presented in Box 1 and Figure 1.

Box 1 **Armed to exit.**

  *Trypanosoma cruzi* has morphological characteristics that help the parasite to rapidly adapt to environmental changes, like the transition from the insect to the mammalian host where it needs to escape from the host immune response. A schematic representation of the structures and organelles of *T. cruzi* is shown in Figure 1.

  **Acidocalcisomes**: These are round organelles that store Ca^2+^, polyphosphates (polyP), magnesium, and other cations. These organelles present pumps and exchangers on their membrane, contributing to the maintenance of pH, cell signaling by the release/capture of Ca^2+^, and osmoregulation in association with the contractile vacuole.

  **Glycosomes**: Exclusively found in Kinetoplastida, these organelles enclose enzymes of the glycolytic pathway, and therefore glycolysis occurs in this dedicated compartment in great contrast with other eukaryotes, where it occurs in the cytosol. Other metabolic pathways, like de novo synthesis of pyrimidine nucleotides, purine salvage, hydrogen peroxide metabolism, etc., also take place in glycosomes.

  **Reservosomes**: Exclusively found in the subgenus *Schizotrypanum*, reservosomes are a pre-lysosomal compartment, where the macromolecules endocytosed by the epimastigotes of *T. cruzi* are stored. Cruzipain, the major cysteine protease of this parasite, is accumulated in this organelle and was found to be crucial for metacyclogenesis (the transition from the replicative epimastigote to the infective metacyclic trypomastigote).

  **Flagellum**: Trypanosomatids present a single flagellum that emerges from the basal body. The flagellum is composed of nine pairs of microtubules disposed around a central pair of microtubules. There is a special area called the flagellar attachment zone on the cell body. The flagellum is present in all stages of the *T. cruzi* life cycle, even in the amastigote form. The flagellum promotes the adhesion to surfaces (a crucial step in metacyclogenesis), motility and control of morphogenesis.

  **Flagellar pocket**: This is a vital structure to *T. cruzi* because all the endocytic activity occurs at the flagellar pocket, except in the epimastigote form where it also occurs in a structure called the cytostome. The flagellar pocket is also involved in exocytosis, cell morphogenesis and immune evasion.

  **Mitochondrion**: *T. cruzi* presents a single mitochondrion that branches throughout the parasite’s body, below the subpellicular microtubules and plasma membrane. Beyond the production of energy, the mitochondrion acts in cell death by apoptosis, and is the main production site for reactive oxygen species (ROS), being trypanothione reductase, an unusual variant of the antioxidant glutathione, is essential for parasite survival.

  **Kinetoplast**: This special structure gives the name for the order Kinetoplastida and refers to the mitochondrial DNA typically found as a disk in epimastigotes and amastigotes, and rounded in trypomastigotes, although always found anterior to the nucleus. The kinetoplast presents a large network of catenated circular DNAs (called kDNA) of two types: maxicircles and minicircles. The maxicircles are 20–40 kb long (depending on strain) and are present in a few dozen identical copies. They encode mitochondrial genes such as rRNAs and subunits of the mitochondrial respiratory chain complexes. Some protein-coding genes are encrypted, meaning that to generate functional mRNAs, the maxicircle transcript must undergo a post-transcriptional modification carried out by guide RNAs (gRNAs) which are mostly encoded by the DNA of the minicircles. Minicircles are present in thousands of copies and are practically identical in size (between 0.5 and 10 kb, depending on strain) but are heterogeneous in sequence.

  **Nucleus**: This is similar to other eukaryotic cells, presenting approximately 2.5 µm, being elongated in trypomastigotes, and rounded in amastigote and epimastigote forms. The nuclear membrane has pores, with continuity between the outer membrane and the endoplasmic reticulum. A typical nucleolus is only seen in the epimastigote form. Chromosomes are difficult to distinguish, as they do not condense at any stage of the parasite’s life cycle.

  **Subpellicular microtubules (SPMT)**: These are the main components of the trypanosomatid cytoskeleton, consisting of α and β-tubulin heterodimers tightly associated with the plasma membrane. SPMT provide the maintenance of cell shape and rigidity, and are associated with organelles (like the endoplasmic reticulum), contributing to the maintenance of the organelle shape and serving as a substrate for organelle locomotion within the cell.

## 3. The Host Immune Response against *T. cruzi* Is Effective, but Not Sterilizing

To establish a persistent infection, *T. cruzi* must strike a balance between causing disease and remaining under the radar of the mammalian immune system. Consequently, this parasite has developed a wide range of mechanisms to evade the immune system, including the expression of various virulence factors (like the ones coded by the multigene families), the establishment of an intracellular replicative niche and the maintenance of reservoirs in certain organs.

In the early moments of infection, the metacyclic trypomastigote forms of *T. cruzi* have two missions: to evade the host’s innate immune system and quickly invade cells to ensure their cycle. One of the first lines of the innate immune response is the complement system, which comprises a set of proteins that are activated by three different pathways, resulting in the lysis of pathogens [44]. The complement system has been shown to play an important role in the recognition of *T. cruzi* metacyclic trypomastigotes and in controlling parasite invasion, although it does not completely eliminate the parasites [45]. *Trypanosoma cruzi* has on its surface a wide range of molecules that interfere both in the initiation of the complement system pathways and in the assembly of C3 convertase. Among the important molecules for resistance to attack by the complement system are calreticulin TcCRT [46], TcCRP [47], TcCRIT [48], GP58/68 [49], and T-DAF [50], which are differentially expressed at different stages and by different strains of the parasite [51]. Furthermore, the induction of the release of extracellular vesicles (EVs) by *T. cruzi* also participates in the inhibition of the complement system. These EVs are lipid bilayer nanoparticles that are secreted by virtually all cells, carrying different biomolecules (proteins, lipids, nucleic acids, etc.) [52]. It was seen that EVs derived from the plasma membrane of host cells in contact with *T. cruzi* are capable of forming a complete C3 convertase and delaying the deposition of the complement system [53], protecting the parasite and transferring resistance to sensible ones [54,55,56]. 

In addition to the humoral components, the innate immune response has a range of cells that participate in the first combat against pathogens and can dictate signals for the adaptive immune response, including macrophages, neutrophils, dendritic cells and natural killer cells (NK). Macrophages act in response to infections and their inflammatory activation exerts cytotoxic effects, mainly via ROS and nitric oxide production. The inflammatory environment at the beginning of the infection leads to macrophage activation. However, *T. cruzi* has strategies to deal with (and even overcome) oxidative environments [57]. The parasite has an elaborate antioxidant system that is based on enzymatic or non-enzymatic molecules to prevent ROS/NO-mediated death and promote greater replication within macrophages. These mechanisms are mainly based on dithiol trypanothione [T(SH)2, N1,N8-bisglutathionylspermidine] and thioredoxin homologue tryparedoxin (TXN) [58]. One of these pathways is the metabolism of trypanothione. This mechanism involves tryparedoxin peroxidases that catalyze the reduction in a broad spectrum of substrates, including hydrogen peroxide (H_2_O_2_), peroxynitrite (ONOO^−^), and organic hydroperoxides (ROOH) [59]. Furthermore, this pathway is identified as a promising target to achieve selective inhibition of the parasite, as it is found exclusively in kinetoplastids [60]. Furthermore, *T. cruzi* contains four iron superoxide dismutases (SODs), which help protect the parasite against the direct cytotoxic effects of O^2−^ and, therefore, inhibit the formation of ONOO^−^ by detoxifying the superoxide radical [61,62]. 

The importance of these *T. cruzi* antioxidant systems is reflected in their success infecting the host. It has been shown that the infection of mice with parasites overexpressing cytosolic tryparedoxin peroxidase leads to increased parasitemia [63]. Zago et al. [64] showed that two strains presenting greater pathogenicity have higher levels of cytosolic and mitochondrial tryparedoxin peroxidases, along with their substrate (tryparedoxin) and iron superoxide dismutase, compared to the low pathogenicity clone. These two strains were also more resistant to exogenous treatment with stable oxidants (H_2_O_2_ and peroxynitrite [ONOO^−^]) and were able to escape intracellular macrophage responses. The enrichment of pathways related to antioxidant defenses may also point to the establishment of chronic infection, as shown by Herreros-Cabello et al. [65], who analyzed the proteomics of two strains. Furthermore, the overexpression of cytosolic superoxide dismutase (Fe-SODB) made these parasites more resistant to macrophage-dependent killing and produced higher parasitemia and parasite burden in the heart tissue of infected mice [66]. In addition to the evident protective function against the macrophage response, these enzymes provide an advantage in the parasite’s resistance against the currently used antitrypanosomal drugs benznidazole and nifurtimox [67].

Dendritic cells (DCs) have special characteristics that allow them to act as professional antigen-presenting cells and are central to the link between innate and adaptive immunity. Immature DCs capture and process antigen and undergo a process of activation and maturation after recognizing conserved molecular patterns associated with pathogens. As an important strategy for subverting the immune response, *T. cruzi* limits the maturation of DCs and leads them to a more tolerogenic profile, reducing the expression of surface molecules (such as MHC, CD80, CD86) and modulating the profile of cytokine release [68,69,70,71]. Therefore, *T. cruzi* overcomes the host’s innate responses and quickly infects host cells where it will continue its intracellular cycle, replicating in the amastigote form. Intracellularly, the parasite can reach incredible numbers of up to 1000 parasites per cell [72] that leads to its rupture, generating trypomastigotes that will infect adjacent cells or enter the bloodstream to infect distant tissues. Therefore, the primary invasion does not awaken the host’s immune system and then gets a “free pass” during the early infection.

After this first round of parasite release and the destruction of host cells, the host’s immune system is exposed to damage-associated molecular patterns (DAMPs) and to pathogen-associated molecular patterns (PAMPs) released from parasites, or it is degraded by the parasite’s products [73,74] triggering inflammation, attracting immune cells to the site of infection, and initiating the adaptive response. The CD8^+^ T cell response is crucial for controlling the intracellular infection [75,76,77,78] and modulating the immune environment [79]. In CD, the induction of an extremely robust, although relatively slow, CD8^+^ T-cell response occurs, with detection of *T. cruzi*-specific CD8^+^ T cells evident only after 8–9 days of infection [80,81].

The main targets of the CD8^+^ T cell response are the parasite antigens exposed at the time of host cell rupture, which mainly include proteins from the trans-sialidases family that cover the surface of *T. cruzi* and are also secreted. Although trans-sialidase molecules are not the only targets of *T. cruzi*-specific CD8^+^ T cells, there appears to be an immunodominance for these epitopes [82,83,84,85]. This immunodominance by trans-sialidases has some drawbacks: TSs have a high variability between strains, based on sequence and expression patterns, and their expression occurs late in the intracellular infection cycle. This way, the parasite has time to replicate before being recognized by the host immune system.

**Figure 2:**
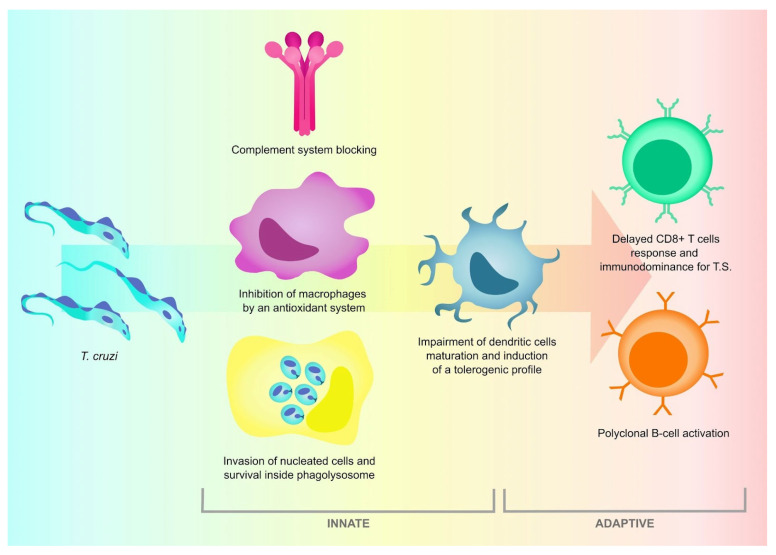
Some of the mechanisms used by *T. cruzi* to overcome the attack of the innate and adaptive immune system.

There is no evidence that *T. cruzi* presents classic antigenic variation, which is the primary immune evasion tool of *T. brucei*. However, the rich diversity of antigenic surface proteins, such as mucins, trans-sialidases and MASPs leads the immune system to a series of spurious and non-neutralizing antibody responses [86,87,88], a mechanism known as smokescreen, which delays the production of high-affinity anti-T antibodies. Added to this non-neutralizing antibody response is polyclonal B cell activation and hypergammaglobulinemia that delays the parasite-specific antibody response [87,89,90,91]. This poor humoral response is crucial for the establishment and progression of infection. Some of the mechanisms used by *T. cruzi* to overcome the attack of the innate and adaptive immune system are schematically represented in Figure 2.

Given that, the success of a CD vaccine would depend on its ability to induce a TH1–mediated immune response [92]. Some strategies have been evaluated in preclinical studies, but face challenges regarding their long-term protection, the variability of strains, and the limitation of extrapolating results from studies in mice, along with the scarcity of investments in this area [93].

After the initial infection, parasitemia reaches its maximum peak (in terms of total number of parasites and tissue dissemination) at around two to three weeks. After high dissemination of the parasite, the immune system is able to progressively reduce parasitic loads in peripheral blood and tissues but is unable to completely eliminate the infection. Now in the progression to the chronic phase of the infection, the parasite can remain in host tissues silently for many years. The development of CD and the mechanisms of persistence in host tissues will be detailed in the next section.

## 4. The Pathogenesis of Chagas Disease: A Story of Persistence, Tropism and Dormancy

### 4.1. Trypanosoma cruzi Persistence Is Crucial for the Development of the Pathology

The pathogenesis of CD comprises an acute phase and a chronic phase. The acute phase, which begins after the entry of *T. cruzi* (classically by the bite of the triatomine vector) is asymptomatic in most cases [1]. When symptoms are present, patients usually exhibit an inflammatory reaction in the skin (chagoma) or conjunctiva (unilateral indurated periorbital lesion known as Romaña’s sign) typically found in endemic areas. A small proportion of patients present other clinical manifestations, such as fever, headache, joint and muscle pain, arrhythmias, lymphadenopathy, and hepatosplenomegaly, among others [94]. 

As the vast majority of individuals infected with *T. cruzi* do not present acute typical signs of infection, i.e., chagoma or Romaña’s sign, patients do not seek health care. This is a particular problem because the medications used in CD (benznidazole and nifurtimox) are more effective in the acute phase [95,96], therefore reducing the chances of a cure. Thus, if left untreated, the symptoms of acute infection will disappear spontaneously over weeks to months and the individual will enter an indeterminate phase of CD. The individuals in indeterminate phase present a positive serological and parasitological test; however, there are no signs and symptoms of the disease, no electrocardiographic changes and a normal-sized heart, esophagus and colon. More than two-thirds of individuals infected with *T. cruzi* remain in the clinically intermediate phase throughout their lives [94].

Approximately 30–40% of patients progress to the chronic phase of CD, which generally appears many years after the initial infection, presenting cardiac, digestive and/or neurological complications. It is not possible to predict which individuals in the indeterminate phase will progress to visceral complications [97], however, new studies have demonstrated that the levels of microRNA-208a (a key factor in promoting cardiovascular dysfunction during cardiac hypertrophy processes of heart failure) appears to be a potential biomarker for predicting the risk of CD progression [98]. 

Chagas cardiomyopathy is present in 20–30% of infected individuals, being a complex disease that includes a wide spectrum of manifestations, ranging from minor myocardial involvement to left ventricular systolic dysfunction, dilated cardiomyopathy, arrhythmias, thromboembolic events and terminal heart failure [99]. The development of cardiomyopathy is related to several pathophysiological processes like the passage of parasites through tissues that leads to a cyclical and slow reaction, resulting in a continuous inflammatory reaction that promotes the death of myocardial cells and their replacement by fibrous tissue over the years [100]. However, it has already been seen that myocarditis can develop even in the complete absence of cardiac parasitism [101]. Thus, it is believed that other mechanisms contribute to the induction of chagasic cardiomyopathy, such as cardiac autonomic dysfunction, microvascular disorders and immune-mediated injury. Genetic mechanisms also appear to play a role in the progression of CD, since Laugier et al. [102] found 4720 genes differentially methylated between patients with Chagas cardiomyopathy and controls, of which 399 were also differentially expressed. Among them were genes related to cardiac electrical conduction, immune response and matrix remodeling.

The gastrointestinal form of CD is less studied, despite its significant occurrence in approximately 10% of infected individuals [9]. In the gastrointestinal form of CD, the esophagus and colon are the most commonly involved segments. The physiological function of these organs depends on the coordination of waves of muscle constriction and relaxation, and the functioning of sphincters; however, in CD, these functions may be impaired, leading to a progressive increase in the diameter of the organs, called megaesophagus and megacolon. One of the causes of this dysfunction is denervation of the myenteric and submucosal plexus by *T. cruzi*, neuronal destruction by inflammatory response and tissue fibrosis [103,104,105,106,107]. Also, *T. cruzi* infection affects the microbiome of the gastrointestinal tract [108] and also causes disturbances in the metabolites of the esophagus and large intestine in the chronic phase, as shown by an in vivo study [109].

Over the years, some theories have attempted to explain the pathology of CD. A heavily discussed idea was that CD was purely an autoimmune disease, based on the assumption that auto-antibodies generated after infection would cross-react with muscle and neural cells (molecular mimicry) causing damage to them [110,111]. Today, it is argued that the persistence of the parasite is necessary to sustain the tissue damage observed in CD. This hypothesis is based on the detection of parasite-derived biomolecules (DNA, antigen) in chagasic heart tissue, the lack of autoimmune reactivity in the absence of concomitant infection, and the efficacy of early antiparasitic chemotherapy [112].

Failure of the host to eliminate the infection leads to the persistence of the parasite in tissues (either through continuous cycles of cellular entry and exit or through sporadic local infections or reactivation of dormant parasites—discussed below), resulting in direct and immune-mediated tissue damage. For example, cardiomyocytes from infected mice exhibited important changes in electrical properties associated with the inflammatory infiltrate and the persistence of the parasite in the tissue [113]. The fact is that *T. cruzi* has many mechanisms to persist in the host, whether by evading the immune response, by lodging itself in privileged tissues, or by altering its replication rate to remain “unreachable” inside cells. Thus, the complex balance between tolerance to infection and response against the parasite outlines the evolution of CD.

### 4.2. Preference or Restriction to a Certain Host Tissue? Factors That Determine the Tropism of T. cruzi

Despite being an ancient disease, it is still not well understood which factors determine the distribution of parasites in tissues during the infection, which can be the most important factor to the parasite’s persistence. The studies on CD indicate that *T. cruzi* tropism appears to be related to factors of both the host (such as genetic background and immune response) and the parasite (infecting strain, route of infection, etc.). 

Some studies point to characteristics of the strains in lodging themselves in certain tissues. For example, Vago et al. [114] investigated the profile of parasites present in the heart and esophagus of CD patients. Interestingly, in the two patients who had cardiac and esophageal involvement, the kDNA signature of the parasites found in the heart and esophagus of the same individual differed, suggesting that there is a differential tissue distribution of genetically diverse populations of *T. cruzi*.

Additional evidence for tissue tropism comes from the results of experimental *T. cruzi* infection, pointing to the variety of infected organs depending on the strain [115,116]. A systematic study of the distribution of intracellular parasites in the organs of mice inoculated with four different strains of *T. cruzi* revealed a high parasitism in the spleen, liver, and bone marrow in the groups inoculated with the Y and Berenice strains, whereas it was almost absent in those inoculated with the CL strain. Also, they have shown that smooth muscle parasitism was significantly greater with strains ABC and Berenice than with Y and CL [115].

In mixed infections, Andrade et al. [117] revealed that some strains have preferred target tissues, both in pure and mixed infections. Interestingly, it has been shown that some strains may have a higher replication rate in some tissues, even though they do not have greater invasion efficiency [118]. This finding, together with new studies, brings even more complexity to the pathogenesis of CD considering the plasticity of the intracellular cycle of *T. cruzi*, as will be discussed in the next session. Mixed infections were evaluated by Franco et al. [119] using two strains that have different virulence profiles: the CL-Brener clone that caused high mortality, severe acute myocarditis and myositis (which was completely resolved in the surviving animals), and the JG strain that caused zero mortality, predominantly focal acute myocarditis, discrete and focal myositis, and a chronic phase with scattered inflammatory foci. The double infection reduced the mortality rate and at the end of the acute phase, the heart exhibited only the JG strain kDNA, while the skeletal muscles and the rectum exhibited only the CL-Brener kDNA. However, in the chronic phase, tropism varied depending on the number of parasites inoculated, indicating that there are many factors influencing the course of CD.

The targeting of *T. cruzi* strains for lodging in certain organs could be determined by the host’s genetic background. Andrade et al. [120] compared the infection of four strains of mice (BALB/c, DBA-2, c57Black/6, and Swiss) by Col1.7G2 and JG *T. cruzi* strains. The tissue distribution of the parasites was identical for BALB/c and DBA-2 mice, but different in C57BL/6 and Swiss mice. As BALB/c and DBA-2 have the same H-2 haplotype (H-2(d)) and C57BL/6 do not (H-2(b)), it is possible that MHC variability is involved in the tissue distribution of the parasite in hosts.

Although many studies identify the distribution of parasites in tissues in vivo, it is still difficult to elucidate the mechanisms by which parasites settle in certain organs. The study by Tonelli et al. [31] revealed that cardiac tropism appears to be influenced by a peptide motif conserved in GP85/trans-sialidases, which interacts with the vascular endothelium with greater affinity/avidity for the cardiac vasculature than for other organs.

Although the determinants of *T. cruzi* tropism are not yet understood, immunological mechanisms are certainly involved. It was seen that *T. cruzi* infection is pantropic during the acute phase, however, as it progresses to the chronic phase, the parasites are restricted mainly to the gastrointestinal tract, with other organs/tissues only sporadically infected. This restriction to certain tissues appears to be limited by the immune system, since chronically infected animals treated with cyclophosphamide, which causes suppression of lymphocytes, change the “restricted” to a “pantropic” phenotype [121]. Despite a highly effective T cell response at the systemic level, incomplete recruitment of T cells to a subset of colonic infection foci occurs, allowing parasites to replicate and remain in the tissue [122]. Consequently, parasites from privileged reservoir sites, such as the digestive tract, can release parasites that seek other less “permissive” sites, such as the heart, resulting in sporadic cycles of cell entry and local immune activation. Furthermore, these studies showed that chronically infected animals developed cardiac pathology even in the absence of a detectable parasite load, with significant levels of diffuse inflammatory mononuclear cell infiltration and fibrosis in the heart [101].

**Figure 3:**
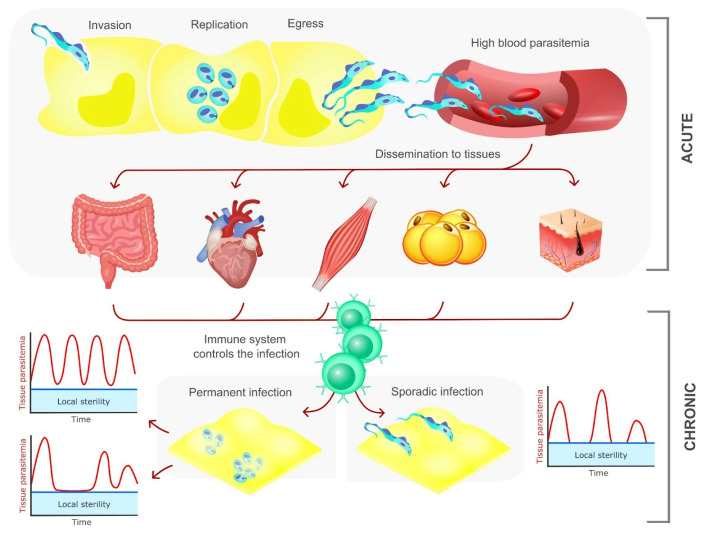
During the acute phase of CD, *T. cruzi* disseminates to all organs of the host. The progress of the immune response restricts the parasites to some organs. The parasite maintains different infection dynamics in tissues, and in some it remains permanent (through constant cycles of reinfection or through persistent amastigotes with a low degree of replication) or in cycles of sporadic infection.

In addition to the heart and gastrointestinal tract, which are of obvious interest due to clinical complications, other tissues may serve as reservoirs for the parasite from which recrudescence may occur during immune suppression. This is the case for adipose tissue and skin. The allocation of *T. cruzi* to adipose tissue has already been demonstrated experimentally and in patients, modifying the secretion of adipokines and altering the metabolic profile of the host [123,124,125,126]. The frequent finding of parasites in the skin of animals infected by *T. cruzi* is of reasonable importance, as it could facilitate the transmission dynamics for the insect vector through the blood meal in the infected host [127]. The distribution of parasites in tissues in the acute and chronic phase and the parasite persistence pathways are schematically represented in Figure 3.

**Figure 4:**
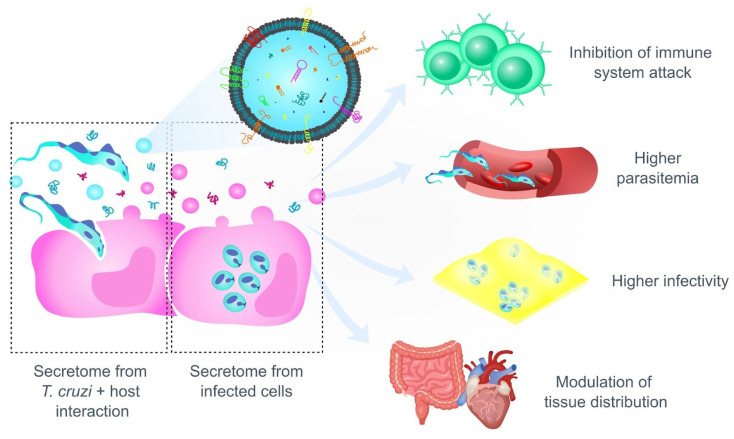
Cellular communication during *T. cruzi* infection. Extracellular vesicles and molecules secreted by the parasite and infected cells modulate infection.

Given the complexity of the parasite’s persistence mechanisms in the chronic phase, it is important to take into consideration techniques that allow the precise mapping of infected cells (instead of homogenized tissues, such as PCR, or macroscale images, such as bioluminescence of organs). In this sense, muscle cells appear to be important reservoirs of *T. cruzi* in the gastrointestinal tract [127]. This “myotropic” characteristic of the protozoan may be related to the easy access to myoglobin as a source of heme [128], and also to this cell type having a high capacity for membrane repair, a mechanism exploited by *T. cruzi* to cause infection [129]. Based on this evidence, the concept of tropism should be taken with caution for CD, as it is a balance of immunological factors and tissue choice. Furthermore, the complex communication between parasite–host through secreted molecules and extracellular vesicles also participates in the pathogenesis of CD (see Box 2 and Figure 4).

Box 2 **Cellular communication participates in the modulation of the infection: the role of the secretome and extracellular vesicles.**

  The microenvironment of infection is of great importance, as *T. cruzi* secretes factors into the environment where infected cells respond by releasing other factors. In eukaryotes, the classical protein secretion pathway occurs via the endoplasmic reticulum (ER)/Golgi pathway using an N-terminal signal peptide. In *T. cruzi*, less than 10% of the proteins found in the secretome contains signal peptides [130]. This suggests that protein secretion via non-classical pathways is of great relevance for this parasite. Secreted proteins can gain access to the extracellular environment by different mechanisms: some proteins anchored in the plasma membrane can have their GPI-anchors cleaved by endogenous phospholipases C and therefore can be released to the extracellular milieu, while others are spontaneously eliminated from the parasite surface in a soluble form or packed in extracellular vesicles (EVs) [26,131].

  Extracellular vesicles are lipid bilayer nanoparticles that mainly comprise exosomes and microvesicles. EVs released by *T. cruzi* contain several virulence factors, such as trans-sialidases, peptidyl-prolyl cis-trans isomerase, oligopeptidases and proteases [130,132,133,134]. In addition to EVs secreted by the parasite, EVs coming from infected host cells or cells in contact with the parasite also participate in communication (Figure 4).

  In the initial stages of infection, contact of *T. cruzi* with host cells in the bloodstream promotes the release of EVs capable of inhibiting the attack of the complement system [53] and increasing the infection of parasites to host cells [54,135,136]. In fact, prior inoculation with EVs released by *T. cruzi* accelerates and increases the mortality rate of infected mice, also triggering more serious cardiac pathology and a greater number of amastigote nests [137]. Similar results are also seen in in vitro studies, in which the addition of EVs is capable of increasing infection in host cells [54,133]. The invasive effects may come from the increase in intracellular Ca^2+^ and the rearrangement of the host cell cytoskeleton caused by EVs [136].

  Not only proteins are packed in EVs: these nanoparticles can also carry other biomolecules, such as nucleic acids and lipids. *Trypanosoma cruzi* EVs carry different RNA contents apparently with a specific targeting to EVs [138,139,140]. It is known that EVs are taken up by host cells and alter their gene expression [135,141,142]. It is not yet known exactly what mechanisms are used to capture EVs or the signals they trigger in host cells, but this exciting field of research can clarify the impact of EVs on parasite–host communication. Furthermore, EVs have great translational biotechnological potential for the diagnosis and production of vaccines. For example, it was found that sera from patients with CD was immunoreactive to proteins present in EVs, placing these particles as a possible biomarker of CD [138]. Regarding vaccines, Gutierrez et al. [143] showed that EVs from the interaction between blood trypomastigotes and bone marrow-derived DCs confer partial protection in animals challenged with lethal *T. cruzi* infection. These results, together with reports on other pathogens such as *Toxoplasma gondii* [144,145], *Eimeria* spp. [146], and *Leishmania major* [147], place EVs as promising therapeutic agents against infectious diseases.

## 5. Perspectives on CD: An Old and Neglected Health Problem

Chagas disease has been classified as a silent and silenced disease, and despite the great scientific efforts to understand and control the disease, it still persists as a public health problem with a global impact due to high morbidity and mortality [10,148]. Despite the increasing state of globalization and urbanization, the occurrence of oral and vertical infection has increased the incidence of CD in several parts of the world, placing it currently as an emerging disease [149,150]. There are several obstacles to diagnosis and treatment, which can affect the number of reported cases, and implies or leads to ineffective public policies [151]. In this context, new diagnostic tools and treatment alternatives are necessary, as well as education that reminds and awakens interest in this silenced disease [152].

### 5.1. Migratory Flows to the United States and Europe from Latin American People Have Increased T. cruzi Infection

The problem of CD may be even greater, since studies have shown that cases are underreported in countries where the disease is not endemic [153]. Global warming represents a possibility of dispersal of traiatomine insects; however, vector control policies have controlled this type of infection. Considering that the main group of people infected or most exposed to CD are young people because they are migrants with the highest mobility [154], and that the main routes of CD transmission in non-endemic areas are congenital or via transfusion, the association between the flow of young Latin migrants and the increased number of CD in non-endemic areas is clear. Vertical transmission is around 3.8%, while seropositivity for CD in high-risk blood donors is 3.9% [155,156]. Recent guidelines for European Countries/The European Economic Area (EU/EEA) public health on screening for infectious diseases in newly arrived migrants unfortunately omit CD [157,158].

### 5.2. Chagas Disease Is Still a Current Health Problem

Due to its latent and chronic nature, *T. cruzi* infection remains an invisible disease in many areas and it is necessary to implement measures that can have a significant impact. The integration of CD diagnosis, treatment and care plans into health services can contribute to the elimination of congenital transmission. Strict implementation of policies to regulate the safety of blood products and organs used for transplantation can ensure complete control of this mode of transmission [159,160]. However, treatment of CD remains a challenge. The only two medications recognized as effective (nifurtimox and benznidazole), discovered between the 1960s and 1970s [161], reduce the duration and clinical severity of CD only when treated in the early steps of the infection (acute phase). Therefore, the treatment is recommended for patients in the acute phase, those at risk for congenital infection, for immunosuppressed patients, and for children with chronic infection [162]. There are other limitations of these medications, like strong side effects that lead to the interruption of the treatment [96]. Despite being discovered 115 years ago, the disease has no effective treatment in the chronic phase, highlighting the fact that CD is a neglected disease facing multiple political, economic, and cultural barriers that influences its scientific research [163]. Therefore, the development of new drugs for the treatment of CD requires strong support, although the lack of investment does not prevent researchers in concentrating their efforts to improve the solubility and bioavailability of current medicines, or to search for new therapeutic compounds [164,165].

New natural and synthetic molecules with antiparasitic activity were found and analyzed, but presented the same disadvantages as nifurtimox and benznidazole [166,167]. Among these compounds, some attracted attention with high in vitro activity, but failed to achieve reproducibility in vivo. New strategies, especially nanotechnology approaches, have shown interesting results seeking to improve the efficiency, activity or performance of nifurtimox and benznidazole or new molecules. Multiple initiatives with different nanoformulations include polymeric nanoparticles, nanoemulsions, liposomes, silver nanoparticles, micelles and liposomes, solid dispersions, and microspheres, among others. Promising and mixed results were found, improving the drug’s efficacy, toxicity, stability and bioavailability [168,169]. Despite these advances, the great challenge and concern regarding the applicability of nanotechnology is to solve treatment problems such as drug targeting, drug resistance mechanisms, and the effects of prolonged release compared to traditional drugs. Most of the work with nanosystems has not explored these points, and the currently restricted clinical trials research should focus on new alternative treatments in detriment to the traditional ones, seeking for more efficient and less toxic drugs [170,171,172].

### 5.3. Between Neglect and Silence: Key Points for Further Research on CD

Despite advances in understanding parasite biology, it is difficult to establish CD control measures. Some of the key points are still under discussion:

- Genetic background of the parasite:

Genetic diversity, post-transcriptional control mechanisms, and other biological peculiarities, make it difficult to manipulate the parasite and to establish control strategies. Moreover, classical genetics and RNA interference strategies do not work in *T. cruzi*, complicating alternatives.

- Complex life cycle:

Several important processes in the parasite’s life cycle are still difficult to understand, from the insect vector, where metacyclogenesis occurs, to the mammalian host. The connection between nutritional depletion, cAMP signaling, nuclear remodeling, post-transcriptional control and differentiation is unknown [173]. Once inside, the parasite evades the immune response and invades tissues to establish the disease. Understanding its mechanism of differentiation, invasion and persistence of infection would enable the design of new drugs or even pesticides to combat CD.

- Silent path between acute and chronic phase of CD:

The complex natural history of the disease hinders its control strategies. Chagas disease has a rapid acute phase, where the parasite avoids the immune response, adapts to the organism, persists in an indeterminate phase for years, and can silently evolve into a chronic disease. Parasite genetic factors and immunological regulations create a clinically variable phenotype that can involve multiple organs in varying degrees of severity, producing distinct forms of CD. A patient-centered and an interdisciplinary approach are needed to adequately address these multiple clinical characteristics in order to improve treatment, and the patient’s quality of life.

- The ineffective treatment for the disease lies in the complexity of the parasite and the lack of public support:

The lack of efficacy of nifurtimox and benznidazole in chronic CD, together with the toxicity and side effects to patients in the acute phase, led to three different search strategies for treatment: the search for new drugs, the improvement of classic drugs using nanomaterials, and the search for drug repositioning with antiparasitic action.

- A neglected disease that reappears, changing the epidemiology and the scenario:

Changes in urban life, vector control achievement in endemic countries, oral contamination in recent decades, and migratory flows in the U.S. and Europe, have dramatically altered the epidemiology of the disease. A new vision of education, disease tracking, diagnosis, and treatment, must lead to public policies for the control of the disease.

- Higher support and investment to improve public health and research on CD:

Public policies must include the prevention, diagnosis and treatment of the disease. Support for basic and applied science is essential to allow better correlation with clinical manifestations, seeking new strategies to control and treat CD.
